# Mechanistic and therapeutic dimensions of DcR3-mediated immunomodulation in sepsis

**DOI:** 10.3389/fimmu.2025.1750066

**Published:** 2026-01-26

**Authors:** Bilal Abbas, Xinrui Lin, Chen Xu, Qi Chen, Jingqian Su

**Affiliations:** Fujian Key Laboratory of Innate Immune Biology, Biomedical Research Center of South China, College of Life Science, Fujian Normal University, Fuzhou, Fujian, China

**Keywords:** apoptosis, DcR3, FasL, immunomodulation, light, sepsis, TL1A

## Abstract

Sepsis is a life-threatening syndrome characterized by dysregulated host-immune responses, progressing through hyperinflammatory and immunosuppressive stages. Decoy receptor 3 (DcR3), a soluble member of the TNF receptor superfamily, serves as an immunomodulator in sepsis. Beyond neutralizing FasL, LIGHT, and TL1A to block apoptosis and inflammatory signaling, DcR3 regulates macrophage polarization, dendritic cell maturation, and immune cell survival through its heparan sulfate proteoglycan-binding domain. Evidence from cellular, molecular, and animal studies highlights its dual role in restoring immune balance by modulating both hyperinflammatory and immunosuppressive phases of sepsis. In this review, we summarize current evidence on DcR3 in sepsis and discuss translational challenges and future directions. Current rodent models lacking the TNFRSF6B gene are limited; however, transgenic mice expressing human DcR3 exhibit both protective and detrimental context-dependent effects. Translational challenges include the pharmacokinetics and immunogenicity of recombinant DcR3, although strategies such as PEGylation, nanoparticle encapsulation, and hydrogel delivery may improve its efficacy. Combining DcR3 with PD-1/PD-L1 inhibitors or immunometabolic agents like metformin and dimethyl itaconate presents promising therapeutic potential. Future research will focus on CRISPR/Cas9 knock-in mouse models, multi-omics mapping of DcR3 signaling, and biomarker-guided dosing. Although no DcR3-targeted clinical trials in sepsis have been conducted, DcR3 remains a precision-targeted immunotherapy with mechanistic and translational pathways; this review delineates key knowledge gaps that must be addressed to enable future clinical application.

## Graphical Abstract

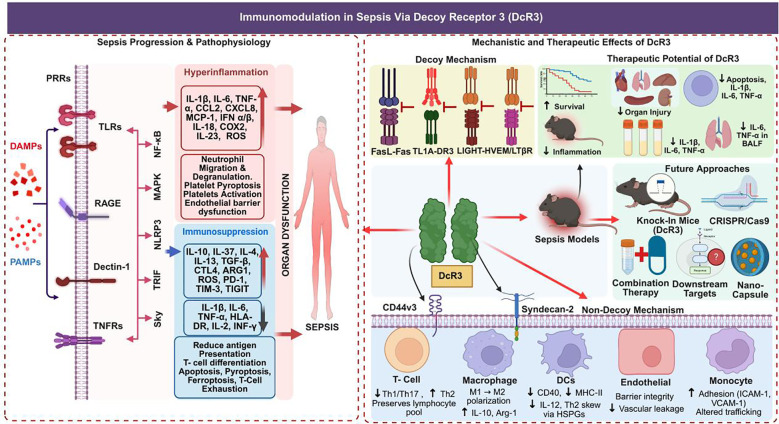

## 1 Introduction

Sepsis is a life-threatening syndrome of acute organ dysfunction caused by a dysregulated host response to infection (1). It represents a major global health emergency due to its rapid progression, high mortality, and lack of effective targeted therapies. The complexity of sepsis arises from its clinical, immunological, and microbiological heterogeneity, as well as its association with various pathogens, including bacteria, viruses, fungi, and parasites (2, 3). The burden is highest in low- and middle-income countries, where limited healthcare infrastructure and diagnostic delays are prevalent (2). The Global Burden of Disease Study estimates that 49 million people develop sepsis annually, accounting for nearly 20% of global deaths, approximately 11 million (2, 4). Despite advances in critical care, sepsis remains one of the leading causes of preventable death worldwide (5).

The pathogenesis of sepsis involves a complex interplay of host-pathogen interactions, with overlapping phases of hyperinflammation and immunosuppression. During the early phase, microbial products like lipopolysaccharide (LPS) induce uncontrolled innate immune activation, marked by cytokine storms, neutrophil recruitment, and endothelial injury. This hyperinflammation leads to hypoperfusion, microthrombosis, and apoptosis. As the condition progresses, immune exhaustion and T-cell apoptosis promote immunosuppression, impair pathogen clearance, and increase the risk of secondary infections and poor outcomes (6, 7).

**Figure 1:**
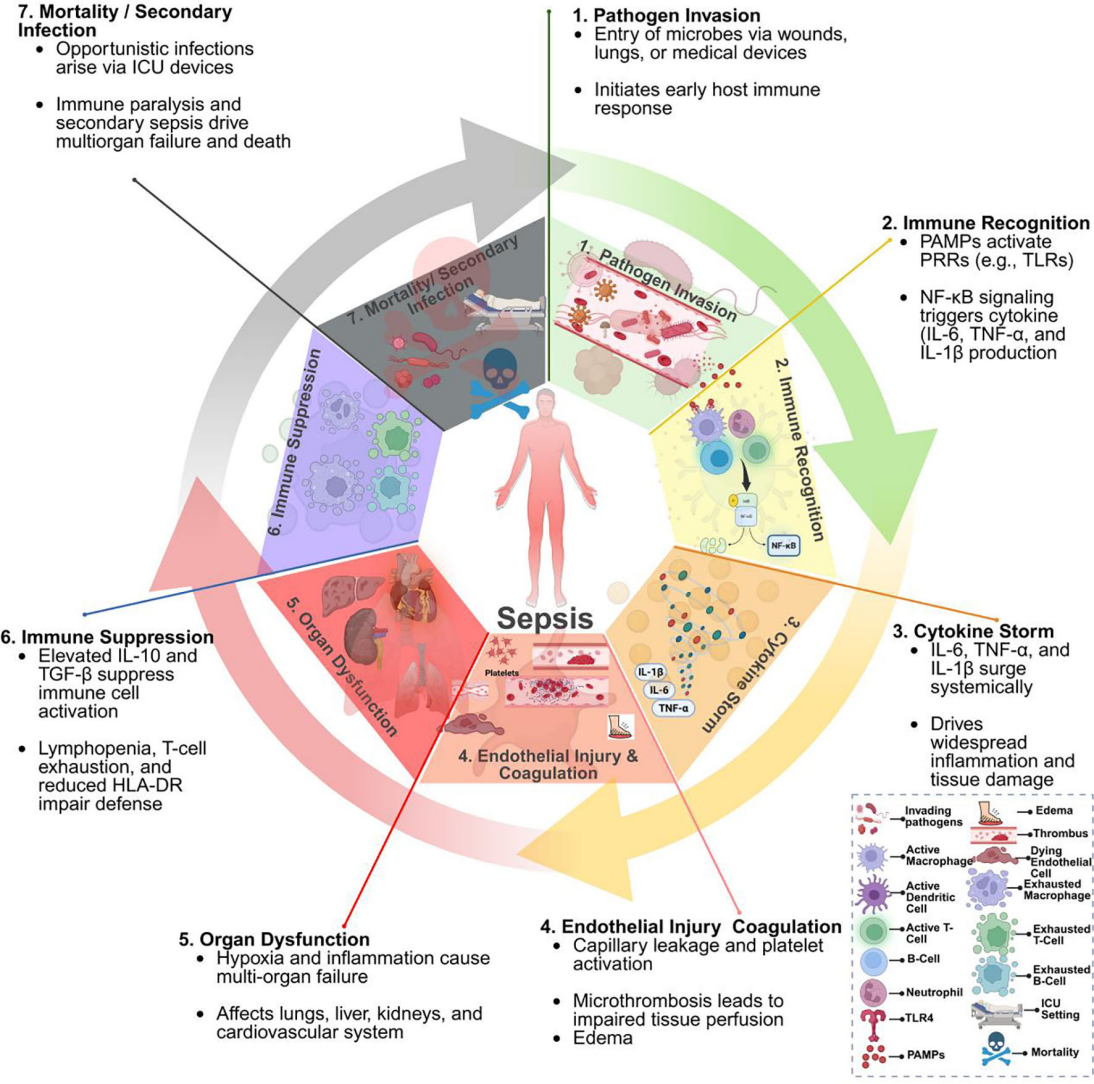
Pathophysiological progression of sepsis. This figure illustrates the sequential stages of sepsis: (1) pathogen invasion; (2) immune recognition via pattern recognition receptors (PRRs), initiating NF-κB-mediated cytokine production; (3) cytokine storm leading to systemic inflammation; (4) endothelial injury and coagulopathy; (5) progression to multi-organ dysfunction; (6) late-phase immunosuppression; (7) increased susceptibility to secondary infections and mortality. The inset highlights immune cell dysfunction over the sepsis timeline. Figure created with BioRender (www.biorender.com).

These immune responses often coexist, rather than occur sequentially, creating the immunological paradox of sepsis (**Figure 1**). This complexity has hindered therapeutic development. Despite over 200 randomized trials of immunomodulatory therapies, no universally effective treatments have emerged. Current management remains largely supportive, involving timely antibiotics, fluid resuscitation, vasopressors, and source control. Even with optimal care, mortality in intensive care settings remains between 25% and 30% (3, 8, 9). These challenges have increased interest in immunomodulatory therapies aimed at balancing pro- and anti-inflammatory responses (9, 10).

Decoy Receptor 3 (DcR3) is a promising immunomodulatory tool for sepsis treatment. Also known as tumor necrosis factor receptor superfamily member 6B (TNFRSF6B), DcR3 is a soluble TNF receptor superfamily (TNFRSF) member that lacks a transmembrane domain, allowing it to circulate in serum (11). First identified in human tumor cells through gene amplification (12), DcR3 binds and neutralizes three TNF superfamily ligands: Fas ligand (FasL), LIGHT, and tumor necrosis factor-like ligand 1A (TL1A), thereby blocking FasL- and TL1A-induced apoptosis and LIGHT-driven inflammation through both classical and non-classical NF-κB pathways. Beyond its decoy role, DcR3 also engages heparan sulfate proteoglycans, including syndecan-2 and CD44v3, to regulate macrophage polarization and T-helper cell differentiation, contributing to immune balance (11, 13).

DcR3 is absent in rodents, limiting conventional knockout studies. However, exogenous or transgenic expression of human DcR3 in mice reduces inflammation, prevents lymphocyte apoptosis, and improves survival in LPS- and CLP-induced sepsis models (11, 12, 14, 15). Clinically, DcR3 levels are elevated in patients with sepsis and correlate with infection severity and biomarkers such as procalcitonin. While this supports its potential as a biomarker, its therapeutic efficacy remains incompletely defined (11, 16). Recent studies in LPS and CLP mouse models, as well as in human and murine inflammatory cells, have shown that exogenous or overexpressed DcR3 suppresses cytokines, limits apoptosis, and improves survival. Collectively, these findings support its dual role as both a diagnostic and therapeutic candidate in sepsis and systemic inflammation (11, 14, 15).

In this review, we evaluate the mechanistic and therapeutic relevance of DcR3 in sepsis, focusing on its decoy interactions with FasL, TL1A, and LIGHT, as well as its broader non-decoy immunomodulatory functions.

## 2 Structure and functions of DcR3

Decoy receptor 3 (DcR3), also known as tumor necrosis factor receptor superfamily member 6B (TNFRSF6B), is a secreted protein belonging to the TNF receptor superfamily. Unlike classical membrane-bound TNFRs, DcR3 modulates immune activity through ligand-neutralizing and receptor-independent mechanisms involved in cancer, autoimmune disorders, and sepsis (11, 12). Encoded by TNFRSF6B on chromosome 20q13.3, the approximately 11 kb gene contains five exons encoding a 300–amino acid protein, including a 29–amino acid signal peptide, producing an approximately 33 kDa mature form. The lack of a transmembrane domain enables DcR3 to be secreted into the extracellular milieu for systemic distribution (11, 13). DcR3 comprises four cysteine-rich domains (CRD1–CRD4) that bind FasL, LIGHT, and TL1A with high affinity, mediating ligand neutralization. These domains resemble the extracellular binding regions of other TNFRSF members. A C-terminal heparin-binding domain (HBD) interacts with heparan sulfate proteoglycans (HSPGs) such as syndecan-2 and CD44v3, promoting non-classical signaling and immunomodulation independent of ligand sequestration (11, 12, 14, 15). Fas ligand (FasL/TNFSF6) is mainly expressed on activated T cells and natural killer cells, where it binds Fas (CD95/TNFRSF6) to induce caspase-dependent apoptosis and activation-induced cell death (11, 17–19). LIGHT (TNFSF14) signals through HVEM (TNFRSF14) and LTβR to provide T-cell co-stimulation and promote vascular and lymphoid inflammation (11, 20, 21). TL1A (TNFSF15) engages DR3 (TNFRSF25) to amplify T-cell responses, particularly Th1 and Th17, and has been implicated in mucosal and autoimmune inflammation. Together, these ligands are key regulators of cell death and inflammatory signaling, which DcR3 can modulate by sequestering them in the extracellular space (11, 22, 23).

DcR3 is absent in rodents because of evolutionary gene deletion (11), limiting the use of conventional knockout models. Its biological roles have been investigated in human DcR3 transgenic mice, which have yielded insights into immune regulation and inflammatory pathology. Although not yet examined in LPS- or CLP-induced sepsis, these systems provide a foundation for future sepsis studies (24, 25). Under physiological conditions, DcR3 expression is minimal but becomes markedly elevated during infection, inflammation, and malignancy. Expression occurs in epithelial and endothelial cells, tumor cells, and macrophages. Increased DcR3 concentrations have been observed in rheumatoid arthritis synovium, inflamed colonic mucosa, and sepsis sera (11, 13).

DcR3 transcription is stringently regulated by inflammatory signaling. Pattern recognition receptors such as TLR4 detect microbial ligands like LPS, activating MyD88- and TRIF-dependent pathways that trigger NF-κB, AP-1, and STAT3, thereby inducing DcR3 expression. In sepsis, TLR4–NF-κB signaling is pivotal (14, 26, 27), while PI3K–AKT activation further enhances transcription and stabilizes mRNA. Pro-inflammatory cytokines (TNF-α, IL-1β, IL-6) also upregulate DcR3, establishing a feedback loop in which DcR3 acts to attenuate excessive immune activation (11).

Epigenetic regulation contributes as well. Promoter demethylation and histone acetylation increase TNFRSF6B accessibility to transcription factors (12), indicating that DcR3 responds to both acute inflammation and chronic immune dysregulation, as observed in cancer and autoimmunity (11, 12). By binding FasL, TL1A, and LIGHT, ligands that normally signal through Fas, DR3, and HVEM/LTβR to drive apoptosis, inflammation, and co-stimulation (22, 26, 27). DcR3 sequesters these mediators, exerting potent immunomodulatory effects relevant to sepsis. Beyond its decoy role, DcR3 interacts with HSPGs to influence macrophage polarization and T-helper cell equilibrium (11).

## 3 Immune dysregulation in sepsis

A central paradox is the coexistence of hyperinflammation and immunosuppression within a single patient, complicating therapy and contributing to the limited success of immune-targeted interventions (7, 28–32). These immune states frequently overlap and evolve dynamically over time, creating marked heterogeneity in host responses.This underscores the urgent need for immunoregulatory therapies and identifies DcR3 as a promising therapeutic candidate.

### 3.1 Hyperinflammation in sepsis

**Figure 2:**
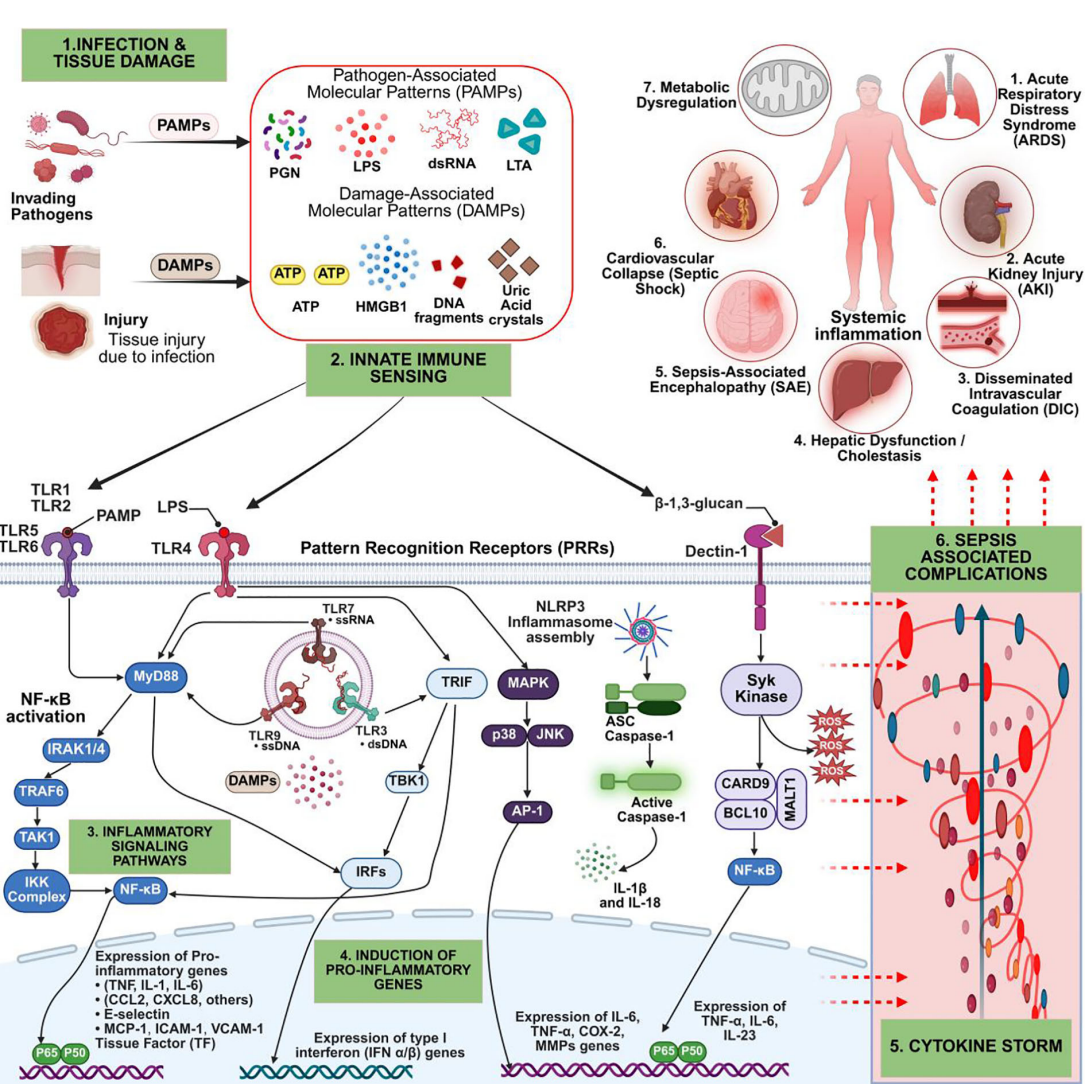
Hyperinflammatory response in sepsis: from pathogen recognition to organ dysfunction. This schematic outlines the steps driving sepsis hyperinflammation: (1) Infection or injury releases PAMPs and DAMPs. (2) These activate PRRs (TLRs, NLRs, Dectin-1) on innate immune cells. (3) PRR signaling via MyD88, TRIF, and Syk triggers NF-κB, IRFs, MAPKs, and inflammasomes. (4) This induces pro-inflammatory cytokines, chemokines, interferons, and adhesion molecules. (5) The cytokine storm causes endothelial injury, oxidative stress, and immune cell recruitment. (6) These events lead to ARDS, AKI, DIC, hepatic dysfunction, cardiovascular collapse, and metabolic dysregulation. Figure created with BioRender (www.biorender.com).

Following infection or sterile tissue injury, innate immune cells recognize pathogen-associated molecular patterns (PAMPs) such as lipopolysaccharides (LPS) and damage-associated molecular patterns (DAMPs), including HMGB1 and ATP, released from necrotic or pyroptotic cells (33, 34). These signals are detected by pattern recognition receptors (PRRs), including Toll-like receptors (TLRs), NOD-like receptors (NLRs), and C-type lectin receptors (CLRs), which initiate downstream signaling cascades that drive inflammation (35–37) (**Figure 2**).

Among PRRs, TLR4 plays a central role by sensing LPS and activating two signaling routes: the myeloid differentiation primary response 88 (MyD88)-dependent and TIR-domain-containing adaptor-inducing interferon-β (TRIF)-dependent cascades. The MyD88 pathway rapidly activates the IKK complex, leading to phosphorylation and degradation of IκBα, permitting NF-κB nuclear translocation and transcription of TNF-α, IL-1β, and IL-6. Concurrently, the MAPK pathway, including p38 and JNK, activates AP-1, which cooperates with NF-κB to amplify pro-inflammatory gene expression (38–40). The TRIF pathway, triggered by endosomal TLR4 or TLR3, activates IRF3 and IRF7, resulting in type I interferon production and integration of innate and antiviral immune responses (34, 41).

Beyond TLRs, other PRRs such as NOD-like receptor NLRP3 play key roles in amplifying inflammation. Upon sensing DAMPs or cellular stress, NLRP3 forms an inflammasome complex with ASC and procaspase-1, leading to caspase-1 activation, maturation of IL-1β and IL-18, and induction of pyroptosis (42–44). HMGB1, released actively or passively during pyroptosis, sustains inflammation through autocrine and paracrine activation of TLR4 and RAGE pathways (45). The CLRs Dectin-1 detects fungal PAMPs and signals through the Syk–CARD9–BCL10–MALT1–NF-κB axis, further promoting NF-κB activation and pro-inflammatory transcription (46, 47). The convergence of these pathways induces a “cytokine storm,” marked by massive TNF-α, IL-6, IL-1β, IFN-γ, and HMGB1 release, leading to vascular leakage, endothelial dysfunction, and disseminated microthrombosis culminating in multi-organ dysfunction syndrome (MODS) (33, 48, 49) (**Figure 2**).

Sepsis-induced hyperinflammation, though initially protective, frequently causes collateral tissue injury and organ dysfunction. Endothelial activation and injury are central, resulting in vascular permeability, tissue hypoperfusion, and microthrombosis, particularly in the lungs, kidneys, liver, and heart (7, 50–52). DAMP-driven NLRP3–caspase-1 inflammasome activation further amplifies inflammation, promoting pyroptosis and barrier disruption (42, 44) (**Figure 2**).

Therapeutically, early attempts to mitigate hyperinflammation with anti-TNF-α antibodies (afelimomab, infliximab), IL-1 receptor antagonists (anakinra), and high-dose corticosteroids failed to improve survival consistently in clinical trials (53, 54). These outcomes are now attributed to sepsis heterogeneity, variations in pathogens, infection sites, host comorbidities, age, and immune-phase timing (3, 55).

### 3.2 Sepsis-induced immunosuppression

Sepsis-induced immunosuppression is a multifactorial phase that contributes to late mortality and secondary infections (56). A defining hallmark is lymphopenia resulting from extensive apoptosis of CD4^+^ and CD8^+^ T cells, B cells, and dendritic cells (DCs) (57). This apoptosis is predominantly caspase-dependent, involving intrinsic (mitochondrial) and extrinsic (death receptor–mediated) pathways such as Fas/FasL signaling and cytochrome c release, which activate caspases 3, 8, and 9 and deplete effector cells (56, 58, 59). Additional forms of programmed cell death, including pyroptosis and ferroptosis, further compromise innate and adaptive immunity (60–62). Reduced expression of major histocompatibility complex class II, particularly monocyte HLA-DR, reflects impaired antigen presentation and correlates with poor sepsis outcomes (63, 64).

**Figure 3:**
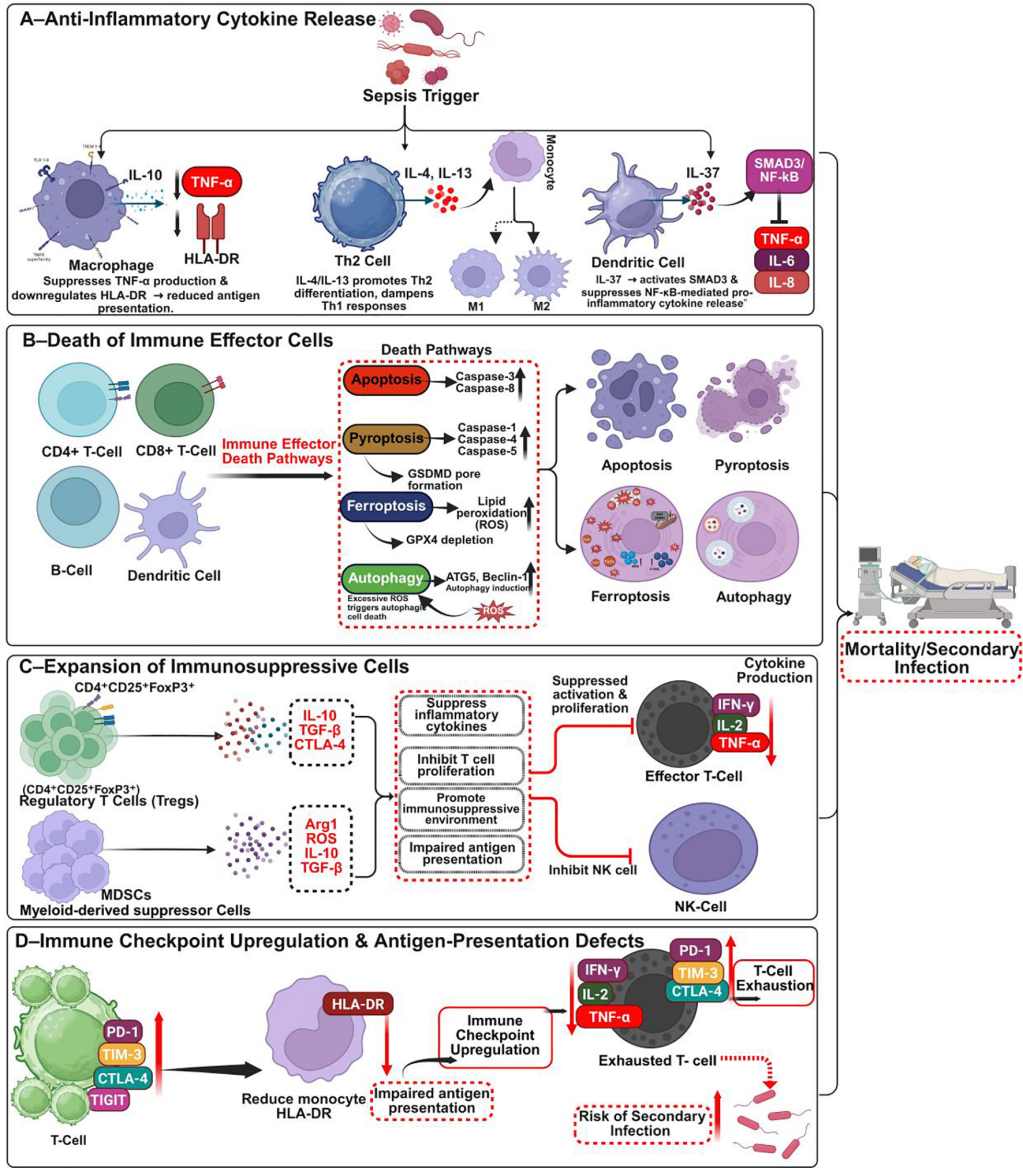
Immunosuppressive mechanisms in sepsis: from anti-inflammation to immune paralysis. This schematic summarizes key immunosuppressive pathways contributing to immune dysfunction and secondary infections in sepsis. **(A)** Anti-inflammatory cytokines (IL-10, IL-4, IL-13, IL-37) suppress HLA-DR and TNF-α/NF-κB signaling, polarizing macrophages, dendritic cells, and T cells toward regulatory phenotypes. **(B)** Widespread apoptosis, pyroptosis, ferroptosis, and autophagy deplete CD4^+^/CD8^+^ T cells, B cells, and dendritic cells. **(C)** Expansion of Tregs and MDSCs suppresses T and NK cell function via IL-10, TGF-β, ROS, and Arg1. **(D)** Upregulation of PD-1, TIM-3, CTLA-4, and TIGIT induces T cell exhaustion, while monocyte HLA-DR downregulation impairs antigen presentation, increasing infection risk. Figure created with BioRender (www.biorender.com).

Concurrently, immune checkpoint pathways become upregulated. T cells exhibit increased expression of inhibitory receptors such as PD-1, CTLA-4, TIM-3, and TIGIT, resulting in exhaustion and decreased cytokine production. These exhausted T cells release less IFN-γ, IL-2, and TNF-α, weakening adaptive immunity and predisposing patients to secondary infections (65, 66). Myeloid cells upregulate PD-L1 while downregulating co-stimulatory molecules, favoring tolerogenic antigen presentation (67). In parallel, endotoxin tolerance develops as monocytes and macrophages repeatedly exposed to PAMPs such as LPS undergo functional reprogramming, with reduced TNF-α and IL-12 but elevated IL-10 and TGF-β expression. This shift culminates in immune paralysis, impairing pathogen clearance and diminishing responsiveness to new infections (68, 69) (**Figure 3**). Importantly, hyperinflammation and immunosuppression frequently coexist in sepsis, complicating therapeutic strategies. Trials targeting hyperinflammation with anti-TNF-α agents, IL-1 receptor antagonists, and corticosteroids largely failed because of suboptimal timing or heterogeneous patient responses (70–73). Likewise, immunostimulants such as IL-7, GM-CSF, and checkpoint inhibitors have shown variable outcomes owing to imprecise phase targeting (74, 75). These challenges underscore the necessity for dual-phase immunomodulatory approaches that balance hyperactive and suppressed immune states according to individual patient profiles.

DcR3 has garnered significant attention for its capacity to modulate both inflammatory and apoptotic signaling. As a soluble decoy receptor, DcR3 binds FasL, TL1A, and LIGHT, preventing their engagement with pro-apoptotic and pro-inflammatory receptors such as Fas, DR3, and HVEM/LTβR. This blockade inhibits FasL-mediated caspase-dependent lymphocyte apoptosis while suppressing TL1A- and LIGHT-induced NF-κB and MAPK signaling, thereby attenuating cytokine release and inflammation. These dual actions position DcR3 as a potent immunomodulator capable of counterbalancing both arms of sepsis-associated immune dysregulation (11, 14, 15). The subsequent sections examine its ligand-specific and intracellular mechanisms in detail.

## 4 Ligand-specific mechanisms of DcR3

DcR3 modulates apoptotic and inflammatory signaling in sepsis primarily through high-affinity neutralization of three TNF superfamily ligands FasL, TL1A, and LIGHT, each of which engages distinct receptor axes (Fas, DR3, and HVEM/LTβR) that contribute to lymphocyte apoptosis, cytokine production, and vascular inflammation in septic immune dysregulation (11, 14, 15). Although DcR3 can also signal via heparan sulfate proteoglycans in a non-decoy manner, the following subsections focus on how it blocks each ligand–receptor pathway to help restore immune homeostasis.

### 4.1 FasL–Fas pathway

The FasL-Fas (CD95) axis is a central regulator of apoptosis, immune responses, and inflammation, particularly in sepsis (17). Fas, a member of the TNF receptor superfamily, is expressed on activated T cells, endothelial cells, and parenchymal tissues, whereas FasL is present on activated CTLs, NK cells, and other immune cells (18). FasL binding induces conformational changes in Fas, recruiting FADD and procaspase-8 to form the death-inducing signaling complex (DISC), which activates caspase-8 (17, 19, 27). This triggers the extrinsic apoptotic pathway, activating caspases-3 and -7, which cleave substrates such as PARP, leading to DNA fragmentation and cell death (19, 76). Beyond apoptosis, Fas engagement activates NF-κB and JNK, promoting IL-6 and TNF-α production (17, 76).

**Figure 4:**
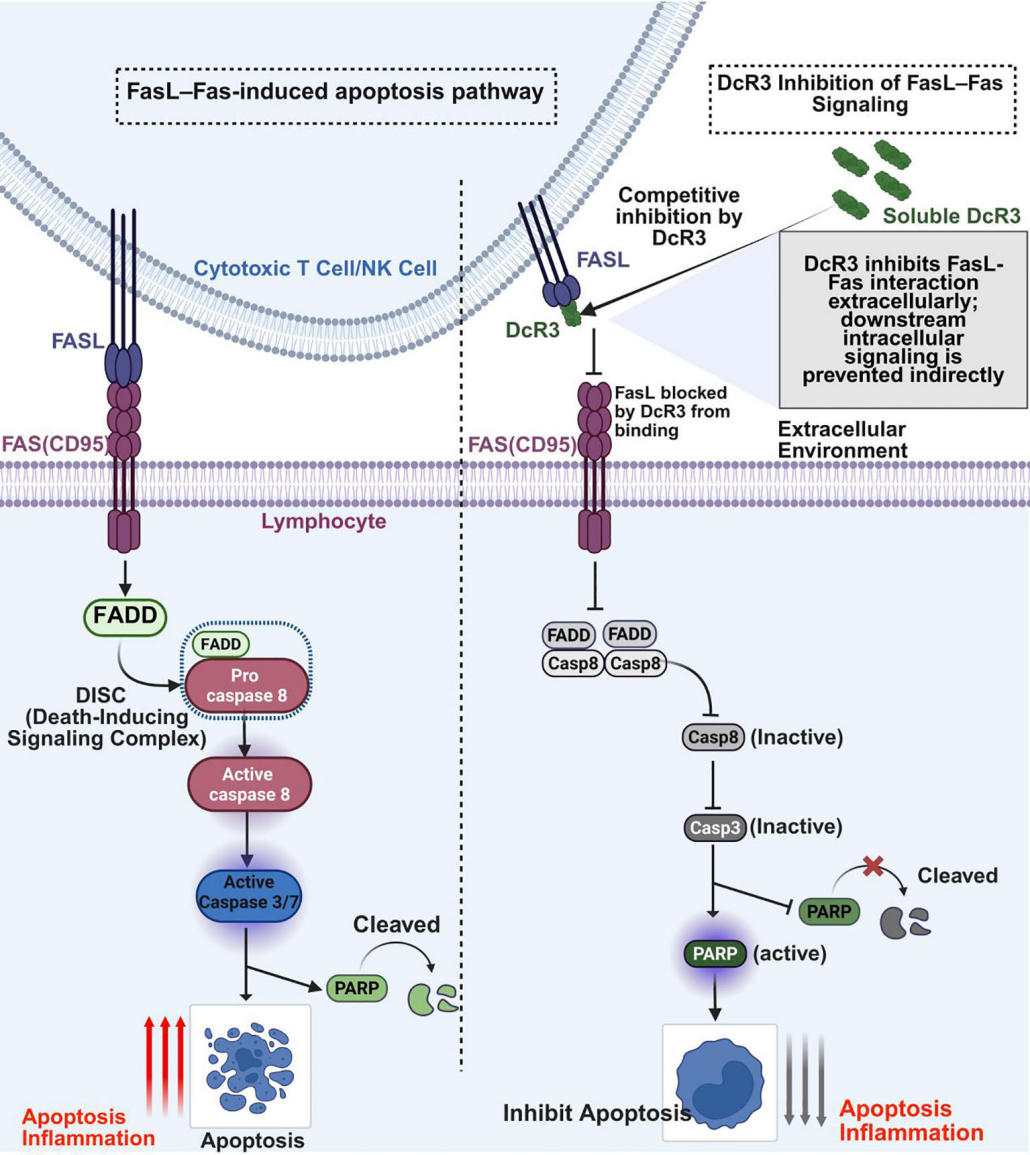
Extracellular inhibition of FasL-Fas signaling by DcR3. This figure shows how soluble DcR3 blocks FasL-Fas (CD95) signaling to prevent apoptosis and inflammation. Left: FasL on cytotoxic T/NK cells binds Fas on lymphocytes, forming the DISC (via FADD and procaspase-8), activating caspases-8/3/7, leading to PARP cleavage, apoptosis, and inflammation. Right: DcR3 binds FasL extracellularly, preventing Fas engagement, DISC formation, caspase activation, and PARP cleavage, thereby suppressing apoptosis and inflammation. The Fas–FasL pathway and DcR3 binding to FasL are well established; the full downstream impact of this interaction on all depicted intracellular events in sepsis is shown here as a conceptual mechanistic model. Figure created with BioRender (www.biorender.com).

In sepsis, Fas–FasL signaling drives immune cell and endothelial apoptosis, contributing to multi-organ dysfunction. FasL-deficient mice subjected to CLP exhibit reduced mucosal lymphocyte apoptosis and improved survival, implicating FasL in sepsis-associated lymphocyte depletion (57, 77–79). Fas activation also induces apoptosis in hepatic endothelial and parenchymal cells, linking Fas signaling to microvascular collapse and tissue injury in septic shock (80, 81) (**Figure 4**).

Studies further demonstrate that CD8^+^ T cells from septic livers upregulate FasL and mediate hepatocyte injury in a FasL-dependent manner, whereas only FasL deficiency, not CD8 deficiency, protects against splenic apoptosis, highlighting tissue-specific FasL pathology (82, 83). Clinically, elevated serum soluble Fas (sFas) concentrations during the first week of sepsis independently correlate with higher SOFA scores and 30-day mortality, emphasizing the prognostic significance of Fas-mediated apoptosis (84).

Therapeutic interventions have targeted this pathway. Administration of DcR3-Fc fusion protein (recombinant DcR3 conjugated to an immunoglobulin Fc domain to enhance stability and half-life) markedly reduces lymphocyte apoptosis and improves survival in CLP-induced sepsis. C57BL/6 mice treated intravenously with DcR3-Fc before and after CLP display significantly reduced thymic and splenic apoptosis, attenuated lung and liver injury, and enhanced 7-day survival compared with controls. *In vitro*, DcR3-Fc pretreatment inhibits FasL-induced apoptosis in human peripheral lymphocytes by suppressing caspase-8 and PARP cleavage (11). More recent findings confirm that DcR3 blocks FasL binding to Fas, reducing systemic inflammation and apoptosis in septic mice (14, 15). Structural analyses show that DcR3 binds FasL with high affinity through conserved TNF ligand recognition sites, efficiently neutralizing apoptotic signaling (85, 86). In cancer research, DcR3 also suppresses FasL-driven apoptosis by downregulating caspase-3/8/9 and enhancing ERK1/2 phosphorylation, revealing broader anti-apoptotic roles across inflammatory and neoplastic conditions (12, 13, 87) (**Figure 4**).

Collectively, these studies highlight the pathological significance of Fas–FasL signaling in sepsis and establish DcR3 as a potential therapeutic candidate capable of preserving immune competence and vascular stability by blocking FasL. However, extrapolating these findings from young inbred CLP mice to human sepsis is challenging, because murine models only partially reproduce the clinical and biological heterogeneity of patients, and many agents that improved survival in mouse sepsis have subsequently failed in clinical trials (20, 21, 88). Moreover, Fas–FasL signaling is essential for activation-induced cell death, peripheral immune tolerance, and elimination of autoreactive or transformed cells, and germline defects in FAS or FASLG in humans and mice cause autoimmune lymphoproliferative syndromes with lymphadenopathy and autoimmunity (89–91).

These observations suggest that prolonged or systemic FasL blockade by DcR3 could theoretically promote lymphoproliferation, autoimmunity, or impaired pathogen and tumor control, emphasizing the need to define dosing, timing, and patient selection carefully in future translational studies. In this context, future investigations should determine whether DcR3 modulates downstream Fas signaling beyond extracellular ligand sequestration and identify therapeutic windows that preserve the homeostatic functions of Fas–FasL while still limiting sepsis-associated apoptosis.

### 4.2 LIGHT–HVEM and LTβR pathways

LIGHT (TNFSF14), produced by activated T cells, NK cells, and immature DCs, is a potent immunomodulator that signals through two primary receptors: HVEM (TNFRSF14) and LTβR. These pathways regulate apoptosis, lymphocyte activation, inflammation, and tissue remodeling (92, 93). LIGHT–HVEM engagement activates the classical NF-κB pathway by recruiting TRAF2 and TRAF5, which stimulate TAK1 and the IKK complex (IKKα, IKKβ, IKKγ/NEMO). This causes IκBα phosphorylation and degradation, releasing the p65/p50 NF-κB dimer to induce transcription of pro-inflammatory cytokines (TNF-α, IL-6, IL-8) and chemokines (CCL19, CCL21), thereby enhancing T cell co-stimulation, monocyte activation, and lymphocyte survival (38, 85, 92).

**Figure 5:**
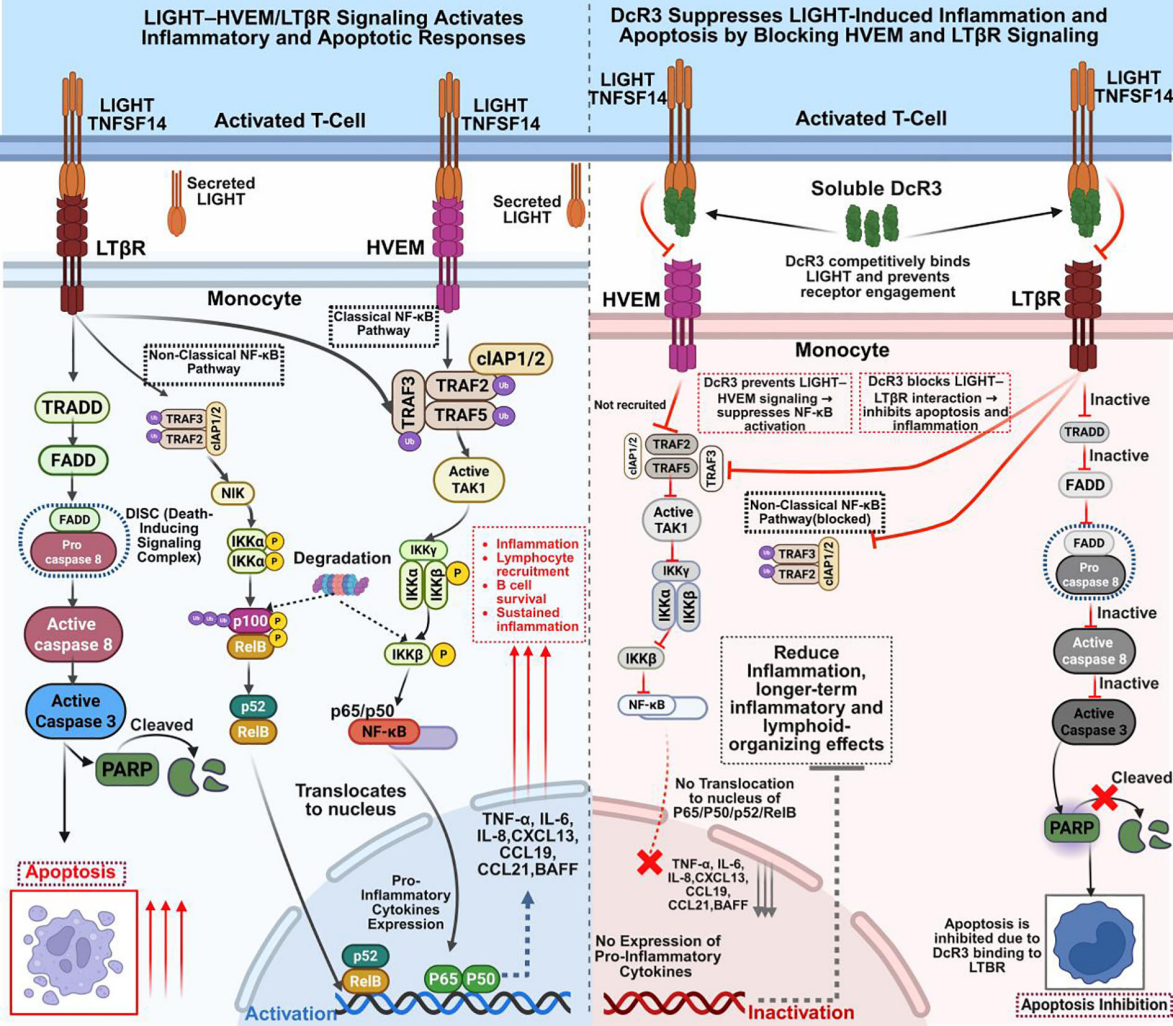
DcR3 extracellularly blocks LIGHT-HVEM/LTβR signaling to suppress inflammation and apoptosis. This figure shows how DcR3 inhibits LIGHTmediated signaling in monocytes. Left: Without DcR3, LIGHT binds HVEM and LTβR, activating classical and non-classical NF-κB pathways via TRAF/TAK1/IKK and inducing pro-inflammatory cytokines (TNF-α, IL-6, IL-8, CXCL13, CCL19, CCL21, BAFF). LTβR also triggers TRADD-FADD-caspase activation, leading to PARP cleavage and apoptosis. Right: DcR3 binds extracellular LIGHT, preventing receptor engagement, adaptor recruitment, NF-κB activation, and caspase signaling—thereby reducing inflammation and cell death. The LIGHT–HVEM/LTβR pathways and DcR3’s decoy binding to LIGHT are supported by experimental data; their combined effect on the entire downstream cascade in sepsis is depicted as an integrative, hypothesis-generating model. Figure created with BioRender (www.biorender.com).

Simultaneously, LIGHT binding to LTβR activates the non-classical NF-κB pathway and apoptosis. It recruits TRADD, TRAF2/3, and cIAP1/2, stabilizing and phosphorylating NIK, which activates IKKα homodimers to process p100 into p52. The resulting p52/RelB complex regulates genes associated with chronic inflammation, lymphoid organogenesis, B cell maturation, and fibrosis (23, 92, 94, 95). The TRADD–FADD axis at LTβR promotes caspase-8 activation and apoptosis, particularly in immune and stromal cells under inflammatory stress (96, 97) (**Figure 5**).

Together, HVEM-mediated (classical NF-κB) and LTβR-mediated (non-classical NF-κB/apoptotic) signaling amplify immune responses. LIGHT induces chemokines such as CXCL13 and BAFF, supporting tertiary lymphoid structure formation and adaptive immunity. However, in sepsis, autoimmunity, and chronic inflammation, excessive activation results in cytokine overproduction, immune infiltration, tissue destruction, and fibrosis (94, 98, 99).

DcR3 neutralizes LIGHT by binding extracellularly at receptor interfaces, thereby blocking its interaction with HVEM and LTβR. Functional studies demonstrate that DcR3 inhibits LIGHT-induced apoptosis and NF-κB activation, while molecular docking reveals strong and stable binding between LIGHT and DcR3 or its SUMO-modified form, confirming DcR3 as a high-affinity decoy (13–15, 23) (**Figure 5**). Although DcR3 suppresses LIGHT-driven inflammation and apoptosis through extracellular sequestration, its direct intracellular effects and any alternative modulatory mechanisms remain undefined, and most functional insights into LIGHT–HVEM/LTβR biology still derive from *in vitro* systems or chronic intestinal/autoimmune models rather than polymicrobial sepsis (89, 100). Because LIGHT can also support tissue repair, barrier integrity, and resolution of inflammation in specific anatomical contexts, sustained systemic LIGHT neutralization by DcR3 could plausibly disrupt beneficial as well as pathogenic responses, underscoring the need for organ-specific and humanized sepsis models to clarify the translational balance of targeting this axis (100).

Although DcR3 suppresses LIGHT-driven inflammation and apoptosis, its actions occur exclusively through extracellular sequestration. The intracellular effects and potential alternative modulatory mechanisms of DcR3 remain to be elucidated.

### 4.3 TL1A-DR3 pathway

TL1A (TNFSF15), also known as VEGI, and its receptor DR3 (TNFRSF25) mediate both inflammatory and apoptotic processes in immune and non-immune cells during sepsis. DR3 is upregulated on activated T cells, endothelial cells, and antigen-presenting cells (APCs) (22, 101). TL1A binding recruits TRADD, initiating two principal pathways: inflammation through the TRAF2/5–RIPK1–TAK1–IKKβ–NF-κB (p65/p50) axis and apoptosis via the FADD–caspase-8–caspase-3/-7 cascade (22, 102).

**Figure 6:**
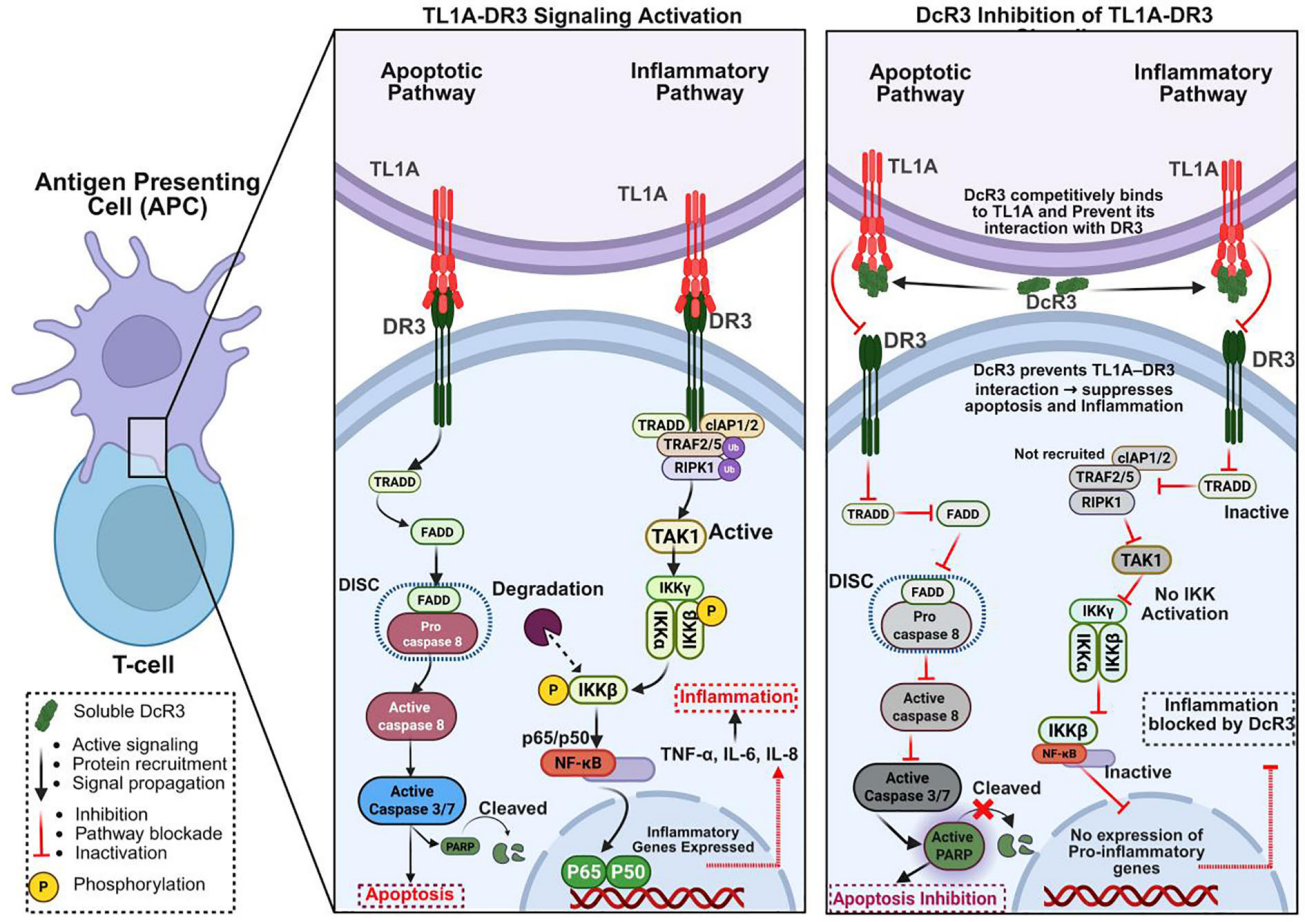
DcR3 extracellularly blocks TL1A-DR3 signaling to inhibit apoptosis and inflammation. This figure illustrates how soluble DcR3 suppresses TL1A-DR3-mediated apoptotic and inflammatory signaling in T cells. Left panel: TL1A binds to DR3, triggering recruitment of adaptor proteins (TRADD, FADD,TRAF2/5, RIPK1) that activate both apoptotic (via caspase-8/3 and PARP cleavage) and inflammatory (via TAK1-IKK-NF-κB) pathways, leading to cytokine production (TNF-α, IL-6, IL-8). Right panel: DcR3 binds TL1A extracellularly, preventing its interaction with DR3. This extracellular inhibition indirectly blocks downstream intracellular signaling, suppressing adaptor recruitment, caspase activation, NF-κB signaling, and ultimately apoptosis and inflammation. The TL1A–DR3 axis and DcR3 binding to TL1A are well characterized experimentally; the overall modulation of all downstream pathways in sepsis shown here should be interpreted as a schematic conceptual model. Figure created with BioRender (www.biorender.com).

In the inflammatory branch, TRADD activates TRAF2/5 and RIPK1, promoting TAK1-driven activation of the IKK complex, IκBα degradation, and NF-κB nuclear translocation. NF-κB subsequently upregulates cytokines (TNF-α, IL-6, IL-8) and chemokines (CCL19/21, CXCL13), driving Th1/Th17 polarization and amplifying immune responses. In sepsis, overactivation results in cytokine storms, leukocyte infiltration, endothelial injury, and multiorgan failure (22, 96). In parallel, FADD recruits caspase-8 to the DISC, triggering caspase-3/7 activation, PARP cleavage, and apoptosis in T cells and endothelial cells—hallmarks of immunosuppression and microvascular damage (101–110). When caspase-8 is inhibited, signaling shifts toward necroptosis via RIPK3–MLKL (111). Although this axis contributes to inflammation in ALI, IBD, RA, and psoriasis, single-cell RNA-seq from ARDS and septic lungs reveals reduced TL1A and DR3 expression with disease progression. Epithelial-specific TL1A or DR3 knockout exacerbates LPS-induced lung injury, demonstrating context-dependent protective epithelial roles (22, 106) (**Figure 6**).

In inflammatory and autoimmune diseases, TL1A–DR3 hyperactivation drives tissue destruction. Agonistic anti-DR3 antibodies aggravate colitis by depleting ILC3s and enhancing neutrophil/eosinophil infiltration through GM-CSF and p38 MAPK (112). Conversely, TL1A blockade with soluble DR3 or antibodies alleviates disease severity in colitis models (103, 105, 112, 113). DR3-deficient mice generate fewer chemokines, show reduced leukocyte infiltration, and are protected against fibrosis and mesothelial injury (106). TL1A–DR3 also contributes to autoimmunity: in RA, TL1A enhances PU.1 expression, promoting Th9 differentiation and IL-9 secretion (114); in psoriasis, TL1A upregulation promotes T cell and neutrophil infiltration, while anti-TL1A therapy reduces inflammation (115). Collectively, these studies identify TL1A–DR3 hyperactivation as a major inflammatory driver, whereas DR3 deficiency confers resistance in multiple disease models (103, 104, 110). Anti-TL1A therapies show therapeutic potential in IBD and airway inflammation (102, 106, 116), though epithelial protection underscores context-dependent effects (110).

DcR3, a soluble decoy receptor, sequesters TL1A and prevents DR3 engagement. Structural analyses confirm high-affinity binding that blocks DISC formation, caspase-8/3 activation, and PARP cleavage (11, 15, 22). By disrupting ligand–receptor interactions, DcR3 prevents assembly of signaling complexes involving TRADD and TRAFs, thereby inhibiting NF-κB activation (24, 115) (**Figure 6**). Experimental data show that DcR3-Fc attenuates anti-CD40–induced NF-κB activity, confirming its inhibitory effect (24). In murine sepsis, DcR3 reduced cytokine production, limited lymphocyte apoptosis, and improved survival without inducing global immunosuppression (14, 15). Although primarily extracellular, whether DcR3 modulates intracellular signaling complexes downstream of DR3 remains uncertain. Moreover, TL1A–DR3 signaling shows context-dependent roles: while pathogenic in chronic intestinal inflammation and fibrosis where TL1A inhibitors and DR3-targeted strategies are now in clinical development, it can also contribute to epithelial repair and barrier homeostasis in preclinical models (22, 101, 103, 107). DcR3-mediated TL1A blockade has not yet been systematically evaluated in human sepsis, so its long-term impact on Th1/Th17 responses, tissue repair, fibrosis, and host defence remains unknown, representing a key translational gap; its dual inhibition of inflammation and apoptosis therefore positions DcR3 as a promising but still experimental immunoregulator whose net clinical benefit will need to be defined in carefully stratified sepsis cohort.

## 5 Non-decoy functions of DcR3

Beyond its established role in neutralizing FasL, LIGHT, and TL1A, DcR3 exerts critical non-decoy immunomodulatory functions through its C-terminal heparan-binding domain (HBD). This domain interacts with heparan sulfate proteoglycans (HSPGs), including syndecan-2 and CD44v3, expressed on immune and stromal cells (11). These interactions are HSPG-specific and independent of ligand binding and can be competitively inhibited by free heparan sulfate (11, 117).

DcR3 influences macrophage polarization by promoting the anti-inflammatory M2 phenotype, characterized by increased CD206 and arginase-1 expression and suppression of pro-inflammatory cytokines TNF-α and IL-6 *in vitro* and *in vivo* (111). DcR3 also modulates dendritic cell (DC) function by downregulating CD40 and MHC-II expression, thereby skewing naïve CD4^+^ T cells toward Th2 differentiation (11, 13). Collectively, these actions limit Th1-driven inflammation while preserving essential host defenses.

DcR3 further regulates monocyte and DC adhesion via CD14-mediated FAK activation and cytoskeletal remodeling, enhancing immune cell retention within inflamed tissues (11, 13, 24). Experimental models demonstrate that DcR3-Fc and DcR3-SUMO reduce IL-1β release, neutrophil infiltration, and macrophage hyperactivation during sepsis. Both forms attenuate lung inflammation and cytokine storms, confirming that non-decoy mechanisms alone can provide tissue protection (11, 13–15).

These non-decoy effects are particularly relevant in sepsis, where DcR3 fosters immune resolution by supporting M2 polarization, modulating Th1/Th2 balance, and enhancing APC function, thereby counteracting immunosuppression without causing systemic hyperactivation. Elevated plasma DcR3 levels in patients with sepsis may represent an endogenous compensatory mechanism to restrain inflammation (11, 118). Overall, DcR3 functions not merely as a ligand trap but as a multifaceted immune regulator whose non-decoy activities hold therapeutic potential for restoring immune balance in sepsis.

## 6 Protective effects of DcR3 across models of sepsis and immune-mediated inflammation

Sepsis remains a leading cause of mortality worldwide, with few pharmacological options to manage its dysregulated immune response (1, 2, 10). DcR3 has emerged as a promising immunoregulator with potent anti-inflammatory and tissue-protective effects (11). In CLP-induced sepsis, recombinant DcR3 improved survival, reduced IL-1β, IL-6, and TNF-α, limited organ injury, preserved gut barrier integrity, and restored microbiota balance by enhancing tight junction proteins and butyrate production. Mechanistically, DcR3 inhibited NF-κB activation and caspase-3 cleavage *in vivo* and in LPS-stimulated macrophages (14). A DcR3-SUMO analog replicated these protective effects, reducing inflammatory cytokines, and achieved a fourfold increase in protein yield without losing ligand-binding activity. Both DcR3-SUMO and native DcR3 improved survival and preserved organ structure, highlighting DcR3-SUMO as a clinically viable, high-yield candidate (15) protective effects of DcR3 across experimental systems are summarized in **Table 1**.

**Table 1:** Experimental studies on the therapeutic role of DcR3 in sepsis and inflammation.

Model/disease	Experimental system	DcR3 form used	Key effects of DcR3	Mechanisms of action	Outcome/findings summary	Reference
CLP- induced sepsis	Mouse model (CLP); RAW264.7 (*in vitro*)	Recombinant human DcR3 protein	↑ Survival, ↓ sepsis score, ↑ body temp stability, ↓ IL-1β, IL-6, TNF-α	Inhibition of NF-κB, TNF, apoptosis pathways; ↑ tight junction proteins; ↓ NLRP3	Broad protection through anti-inflammatory and barrier-restorative effects	(14)
LPS-induced sepsis	Mouse model; THP1, RAW264.7	DcR3-SUMO analog	↑ Survival, ↓ IL-1β, IL-6, TNF-α; preserved tissue	Retained ligand-binding (TL1A, LIGHT, FasL); ↑ protein yield; histological protection	High-yield therapeutic analog with preserved bioactivity	(15)
TD antigen–induced humoral immunity	DcR3-Tg mice (CD68); ex vivo B cell assays	DcR3-Fc and Tg DcR3	↓ Ag-specific IgM, IgG, ASCs; suppressed Igh and Xbp1; ↓ NF-κB	NF-κB inhibition Xbp1 downregulation Impaired plasma cell differentiation	Reduced serum antibodies Decreased Ig secretion XBP1 restoration partially rescues transcription Indicating multiple inhibitory targets	(24)
Sepsis (macrophage polarization)	THP-1 → macrophages (PMA) + LPS	DcR3 overexpression	↓ TNF-α, ↑ CD163, ↑ IL-10; M2 shift	miR-148b targets DcR3; DcR3 reverses miR-148b effects	DcR3 promotes polarization and anti-inflammatory response	(112)
IL-1α–induced osteoclastogenesis	Murine BMMs & RAW264.7 (RANKL + IL-1α)	Recombinant DcR3	↓ Osteoclasts, ↑ apoptosis	ROS ↑ → FasL, IL-1α, IL-1ra induction; IRAK4 activation	DcR3 inhibits osteoclastogenesis, with potential in inflammatory bone loss	(113)

Recent studies broaden DcR3’s therapeutic scope beyond its established immunomodulatory roles in T cells, macrophages, and DCs to include regulation of humoral immunity and bone remodeling. A 2025 study demonstrated that CD68–DcR3 transgenic mice and *in vitro* DcR3-Fc treatment attenuated T cell–dependent antibody responses by transiently reducing germinal center B cells and antibody-secreting cells, accompanied by decreased expression of XBP1 and secretory immunoglobulin heavy chain (Igh) transcripts. These changes led to sustained reductions in antigen-specific IgM and IgG levels (24). This mechanism introduces a humoral regulatory dimension to DcR3’s functions, expanding its relevance to sepsis, where dysregulated antibody responses worsen disease progression.

**Figure 7:**
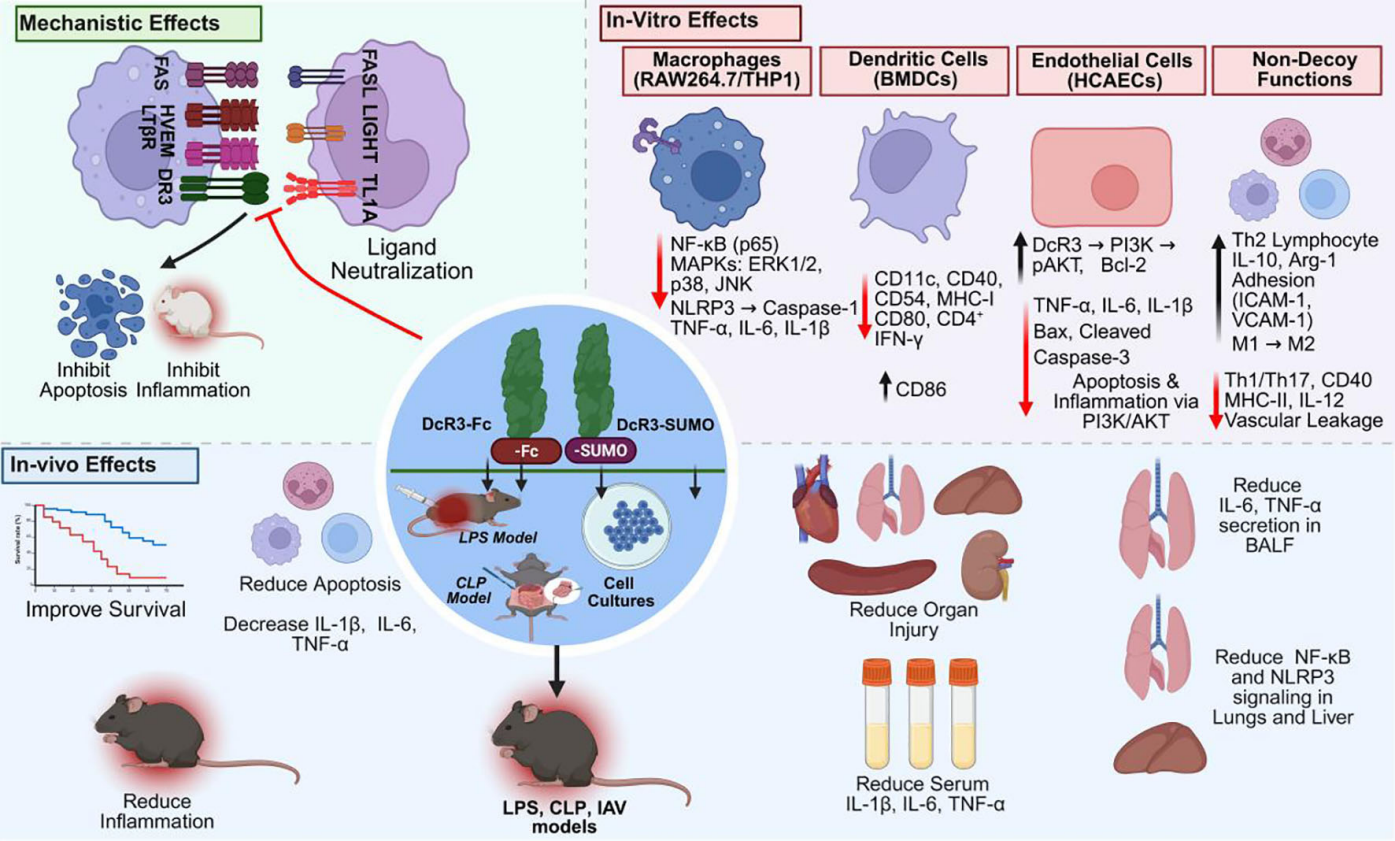
Therapeutic mechanism of DcR3 in experimental sepsis and inflammation. DcR3-Fc and DcR3-SUMO neutralize FasL, LIGHT, and TL1A, blocking their receptors (FAS, HVEM, DR3, LTβR) to exert anti-inflammatory and anti-apoptotic effects. DcR3 suppresses NF-κB, MAPK, and NLRP3 in macrophages, reduces dendritic cell co-stimulation, and inhibits endothelial apoptosis via the PI3K/AKT pathway. In LPS-, CLP-, and IAV-induced sepsis models, DcR3 improves survival, lowers cytokines, limits organ injury, reduces lymphocyte apoptosis, and promotes M2 macrophage polarization. Figure created with BioRender (www.biorender.com).

Additionally, DcR3 inhibited IL-1α–driven osteoclastogenesis in murine bone marrow macrophages and RAW264.7 cells, reducing osteoclast formation and bone resorption through ROS accumulation, FasL induction, and IL-1ra upregulation (119). The cellular and molecular pathways through which DcR3 exerts these protective effects across immune and endothelial compartments are illustrated in **Figure 7**.

Collectively, findings from infectious, autoimmune, and sterile inflammatory models reveal the capacity of DcR3 to modulate immune activity, limit cytokine-driven tissue injury, and preserve organ function, underscoring its potential for translational development in sepsis and systemic inflammatory syndromes.

## 7 Challenges and future perspectives in DcR3-based therapeutics

Although DcR3 shows considerable promise as a therapeutic immunoregulator in sepsis and other inflammatory conditions, several critical challenges must be resolved before clinical translation can be achieved.

A major limitation is the absence of DcR3 (TNFRSF6B) in rodent genomes, which restricts investigation in standard murine models (11). To overcome this, transgenic mice expressing human DcR3 under broad promoters have been developed. For instance, β-actin promoter–driven expression induced systemic DcR3, resulting in a Th2-biased immune profile characterized by elevated IL-4/IL-10 and reduced IFN-γ/TNF-α (24). Similarly, CD68 promoter–driven expression in macrophages polarized tumor-associated macrophages toward the M2 phenotype, characterized by increased Arg-1 and CD206 and reduced inflammatory cytokines, findings relevant to sepsis outcomes (13). DcR3-expressing DCs also mitigated autoimmunity and improved islet graft survival in T1D transplantation models (120).

Although these models demonstrate the anti-inflammatory, anti-apoptotic, and immunomodulatory functions of DcR3, they rely on artificial promoters such as β-actin or CD68, which produce ectopic expression and non-physiological protein levels, diverging from the tightly regulated expression observed in humans (121).

An improvement may come from CRISPR/Cas9-mediated knock-in of human TNFRSF6B into mice under its native regulatory elements, better reflecting physiological dosage, tissue specificity, and inducibility (122, 123). This approach parallels humanized strains such as MITRG/MISTRG, in which murine cytokines are replaced with human orthologs (124). Unlike conventional transgenics, CRISPR knock-ins prevent random integration and overexpression artifacts (122, 124, 125). Such models would enable precise study of tissue- and context-specific DcR3 activity in sepsis, maintaining temporal control consistent with disease states such as pancreatitis (126).

Recombinant DcR3 variants (Fc-fusion, SUMO-tagged) enhance protein stability and yield compared to native DcR3 (14, 15). However, the native protein exhibits rapid clearance and limited tissue accumulation, constraining sustained anti-inflammatory efficacy. Challenges remain in optimizing dosage, delivery methods, and minimizing immunogenicity (127). For other cytokines and protein therapeutics, several delivery platforms have been developed to extend serum half-life and improve targeting, including PEGylation, which has been used to generate long-acting IL-10 conjugates with improved pharmacokinetics in cancer immunotherapy (128); nanoparticle encapsulation, which protects proteins from degradation and enables tissue-specific release (129); and hydrogel depots, which sustain local delivery in inflammatory arthritis models (130). While these strategies have been successful for other cytokines and biologics, they have not yet been applied to DcR3 in sepsis models. A recent dual-responsive magnetic nanoparticle decorated with anti-DcR3 antibodies efficiently targeted DcR3-expressing hepatocellular carcinoma lesions in mice and enhanced local bioactivity, illustrating that DcR3-related epitopes can be exploited by advanced nanocarriers outside the sepsis context (131). Such magnetically guided and PEGylated delivery systems highlight the potential of combining DcR3 with advanced nanocarrier technologies to improve stability, targeting, and therapeutic efficacy in complex inflammatory conditions such as sepsis.

Future studies should investigate whether analogous PEGylated or nanoparticle-formulated DcR3 constructs can safely improve pharmacokinetics and tissue targeting in sepsis, while rigorously evaluating immunogenicity and off-target effects.

DcR3 neutralizes FasL, LIGHT, and TL1A, thereby blocking Fas–FADD–caspase-8 apoptosis and TL1A–DR3–NF-κB/JNK inflammatory signaling (11, 14, 15, 24, 132). Its heparan sulfate proteoglycan-binding domain mediates non-decoy effects on dendritic cell maturation, macrophage polarization, and immune cell survival (11). However, the downstream interactors and intracellular signaling events of DcR3 in sepsis remain poorly defined. Comprehensive mapping through integrated methods, including bulk and single-cell RNA sequencing, proteomics, phosphoproteomics, and spatial multi-omics, will be crucial for identifying pathway-specific effectors and regulatory nodes of DcR3 activity (11, 133–136). Furthermore, emerging evidence suggests that DcR3 may influence gut microbiota composition and short-chain fatty acid production in sepsis (14); however, the mechanistic basis of these interactions and their contribution to therapeutic efficacy remain to be systematically investigated.

Given the biphasic immune response in sepsis, the timing of DcR3 administration is critical (137). Early treatment during hyperinflammation attenuates cytokine storms, tissue damage, and mortality (14, 15). Biomarker-guided dosing approaches using parameters such as IL-6, HLA-DR, or lymphocyte counts may optimize therapeutic windows by initiating DcR3 therapy during hyperinflammation and withdrawing treatment as immunosuppression develops (138).

To date, no clinical trials of DcR3 in sepsis have been conducted (137). Translational progress will require comprehensive toxicological, pharmacokinetic, and dose-ranging preclinical studies to support IND submission. Phase I trials should evaluate safety, immunogenicity, and biomarker responses in carefully selected subgroups exhibiting excessive inflammation or immune dysregulation. Patient selection could mirror precision strategies successfully used in anti-TNF therapy for individuals with elevated IL-6 (138), IL-7 (139), or PD-1 inhibitor levels to reverse sepsis-induced lymphopenia (140, 141).

DcR3 may achieve enhanced efficacy when combined with other immunoregulators. Co-administration with PD-1/PD-L1 inhibitors may attenuate hyperinflammation while reversing T-cell exhaustion (14, 15, 140, 141). In murine models, dual inhibition of IL-6 and PD-1 improved pathogen clearance and reduced tissue injury and lymphocyte apoptosis (142). Combination with immunometabolic agents such as metformin or dimethyl itaconate may restore immune equilibrium by reprogramming macrophages through Nrf2 activation (143, 144). Adjunctive use of natural compounds, including aloin and fucoxanthin (145), improved survival, reduced cytokine levels, modulated microbiota, and suppressed NF-κB/NLRP3 signaling in CLP models (146). Su et al. (147) further demonstrated that aloin combined with TIENAM significantly improved survival and reduced inflammation in CLP-induced septic mice while modifying peritoneal microbiota composition (147).

Future DcR3-based therapies will likely depend on physiologically relevant gene models, precision biomarker-guided dosing approaches, and incorporation into multimodal regimens combining immune regulation, infection control, and tissue protection to optimize outcomes in sepsis (136).

## 8 Conclusion

DcR3 is a distinct immunoregulatory molecule in sepsis, acting both as a soluble decoy receptor that neutralizes FasL, LIGHT, and TL1A, and as a non-decoy modulator that regulates macrophage polarization, dendritic cell maturation, and immune cell survival. This dual functionality positions DcR3 at the intersection of immune activation and suppression, emphasizing the importance of precise therapeutic timing and administration. However, translational progress is limited by species-specific expression barriers, suboptimal pharmacokinetics of recombinant proteins, and the lack of clinical trials. Among recombinant variants, DcR3-SUMO has emerged as a promising candidate for clinical translation, demonstrating a fourfold increase in protein yield while retaining full ligand-binding activity and therapeutic efficacy in LPS-induced sepsis models (15). Its enhanced stability and manufacturability address key pharmacokinetic limitations of native DcR3, positioning DcR3-SUMO as a lead candidate for future preclinical toxicology studies and early-phase clinical trials. Biomarker-guided patient stratification using IL-6, HLA-DR expression, or lymphocyte counts may further optimize therapeutic windows and improve trial outcomes (131). Advancing DcR3 toward clinical application will require physiologically regulated knock-in models, detailed downstream signaling characterization using multi-omics technologies, and integration into biomarker-guided, phase-specific therapeutic strategies. Whether as a monotherapy or in combination, DcR3 demonstrates strong potential to restore immune homeostasis in sepsis without compromising host defense.
